# Non-Primate Lentiviral Vectors and Their Applications in Gene Therapy for Ocular Disorders

**DOI:** 10.3390/v10060316

**Published:** 2018-06-09

**Authors:** Vincenzo Cavalieri, Elena Baiamonte, Melania Lo Iacono

**Affiliations:** 1 Department of Biological, Chemical and Pharmaceutical Sciences and Technologies (STEBICEF), University of Palermo, Viale delle Scienze Edificio 16, 90128 Palermo, Italy; 2 Advanced Technologies Network (ATeN) Center, University of Palermo, Viale delle Scienze Edificio 18, 90128 Palermo, Italy; 3 Campus of Haematology Franco e Piera Cutino, Villa Sofia-Cervello Hospital, 90146 Palermo, Italy; elenabaiamonte77@gmail.it (E.B.); loiaconomelania83@gmail.com (M.L.I.)

**Keywords:** FIV, EIAV, BIV, JDV, VMV, CAEV, lentiviral vector, gene therapy, ophthalmology, zebrafish

## Abstract

Lentiviruses have a number of molecular features in common, starting with the ability to integrate their genetic material into the genome of non-dividing infected cells. A peculiar property of non-primate lentiviruses consists in their incapability to infect and induce diseases in humans, thus providing the main rationale for deriving biologically safe lentiviral vectors for gene therapy applications. In this review, we first give an overview of non-primate lentiviruses, highlighting their common and distinctive molecular characteristics together with key concepts in the molecular biology of lentiviruses. We next examine the bioengineering strategies leading to the conversion of lentiviruses into recombinant lentiviral vectors, discussing their potential clinical applications in ophthalmological research. Finally, we highlight the invaluable role of animal organisms, including the emerging zebrafish model, in ocular gene therapy based on non-primate lentiviral vectors and in ophthalmology research and vision science in general.

## 1. Introduction to Non-Primate Lentiviruses

In the biomedical field of gene therapy, retroviridae-based vectors currently represent the most successful vehicles for delivery of functional gene units in vivo [1]. According to the International Committee on Taxonomy of Viruses [2], the Retroviridae family encompasses an ever-growing list of different species officially classified into two subfamilies, Orthoretrovirinae and Spumaretrovirinae, each including six and five genera, respectively. Lentiviruses are members of the Orthoretrovirinae subfamily, offering distinctive advantages for clinical applications: they can stably infect cells regardless of their proliferation status, showing no immunogenicity in vivo [3,4,5].

These viruses acquired their *lenti* (in Latin meaning *slow*) etymological prefix due to the protracted incubation period elapsing between the initial infection and the onset of the disease, usually lasting for months or even years.

Molecular phylogenetic studies coupled to virus–host specificity indicated that lentiviruses probably originated in non-primate mammals, and that they can be split into two major classes, viz primate and non-primate lentiviruses [6,7]. The former class includes viral species such as the human immunodeficiency virus (HIV), while the non-primate lentiviral class comprehends the prototype visna-maedi virus (VMV), as well as the related caprine arthritis encephalitis virus (CAEV), the equine infectious anaemia virus (EIAV), the bovine immunodeficiency virus (BIV), and the more recently described feline immunodeficiency virus (FIV) and Jembrana disease virus (JDV) (Table 1). By virtue of their marked genomic similarities, VMV and CAEV have been recently reassigned to a single quasi-species designated small ruminant lentiviruses (SRLVs) [8,9,10], while JDV is considered a subtype of BIV capable of specifically infecting Bali cattle [11,12].

## 2. Morphology and Genome Organization

The morphology and genome organization of all lentiviruses are similar in many aspects. The lentivirions have been described as slightly pleomorphic spherical-shaped particles with diameters of approximately 100 nm, containing a diploid genome that consists of two single-stranded positive-sense RNA molecules [25,26]. The viral genome is packaged by the nucleocapsid proteins and bound to the reverse transcriptase, integrase, and protease viral enzymes, forming an isometric core frequently shaped like a truncated cone. This internal structure is then encased in a proteinaceous shell formed by capsid proteins, and in turn surrounded by matrix proteins that interact with a lipid envelope. The envelope surface appears rough due to the presence of regularly dispersed projections of about 8 nm, which incorporate the two viral envelope transmembrane and surface glycoproteins.

The genome is organized from the 5′- to the 3′-end into a common modular structure consisting of the three primary *gag*, *pol*, and *env* genes. These genes encode the structural proteins providing the architecture of the virion, the reverse transcriptase/integrase/protease enzymes, and the envelope glycoproteins, respectively. Other genetic characteristics common in lentiviruses are some *cis*-regulatory sequences such as the RNA packaging signal required for genome encapsidation in virions [27], the polypurine tract required for reverse transcription [28], and the two long-terminal repeats [29].

A small set of accessory genes is differentially distributed among non-primate lentivirus species, and it accounts for most changes in molecular organization that differentiate them (Table 2). 

These accessory genes are involved in the regulation of viral replication, assembly, and pathogenesis. Among them, the *rev* gene encodes a protein involved in nuclear export of viral genomic RNA [30]. Rev is the most functionally conserved accessory protein within non-primate lentivirus species, although the Rev protein of FIV bears a quite divergent non-consensus nuclear export signal [31,32].

All non-primate lentiviruses, except EIAV, contain the *vif* gene situated between *pol* and *env*. Though it is categorized as an accessory gene, *vif* encodes a protein that is required for viral infection and propagation, being involved in counteracting the antiviral activities of cellular APOBEC3 cytidine deaminases [33,34,35].

An additional pair of accessory genes consists of *tat* and *orfS*, both encoding regulatory proteins that serve to activate transcription of the lentiviral genome. More specifically, the former protein, which is related to the HIV homonym, acts as a strong transactivator by binding a stem-loop recognition element in the long terminal repeat (LTR) of EIAV, BIV, and JDV [36,37]. However, Tat proteins from SRLVs lack a transactivation function, while the FIV genome does not contain a *tat* gene at all [38,39]. Regarding the OrfS protein, which is called Orf2 in FIV, it is a weak activator of viral transcription that acts by binding to AP1 sites in the LTR of SRLVs and FIV [40,41,42].

Finally, there are also some accessory genes of currently undefined function. In particular, only BIV encodes the two small Vpy and Vpw proteins from the *vif* gene sequence [30,43], only BIV and JDV possess the *tmx* gene situated at the 3′-end of *env* [11,22], and only the EIAV contains the *s2* gene in the central region of its genome [44,45]. 

Beyond the accessory genes, one notable difference between the two mentioned classes of viruses is that all non-primate lentiviruses, except the BIV/JDV lineage, contain a region of the *pol* gene encoding a deoxyuridine 5′-triphosphate nucleotidohydrolase (dUTPase), whereas the primate counterparts lack such a genetic trait [46]. It has been shown that dUTPase has a central role in facilitating productive viral replication in post-mitotic cells, possibly by minimizing misincorporation of potentially mutagenic dUTP into the proviral DNA [47,48]. The genome of the BIV/JDV viruses also possesses an insert in the same region of *pol*, but the sequence similarity is low compared to the primate counterparts, and the region seems to be no longer capable of encoding a functional dUTPase. However, despite the lack of catalytic activity, the encoded protein is critically required for BIV/JDV replication [49]. 

## 3. Life Cycle

The general lentivirus replication cycle starts with the engagement of a specific cellular receptor by the surface viral envelope glycoprotein. Target cell receptors for most of the non-primate lentiviruses remain to be determined. To date, all we know is that EIAV and FIV recognize two distinct members of the tumour necrosis factor receptor family [50,51], and that FIV requires the CXCR4 chemokine coreceptor [52,53].

Whatever the mechanism of ligand binding, the glycoprotein–receptor interaction determines the viral tropism towards specific cell types of the host, allowing the fusion between the cell and virus lipid membranes. This, in turn, results in the release of the viral core particle into the cytoplasm of the target cell, whereas the local dNTP concentration triggers the retro-transcription to cDNA of the viral genome [54]. Lentiviruses use a host cell tRNA^Lys^ molecule to prime reverse transcription of each of the two genomic RNA copies [55,56]. While this process is running, it induces the reorganization of the viral core particle, with progressive shedding of capsid protein units. Next, the partially dismantled core particle is actively transported to the cell nucleus through a nuclear pore, and the virus cDNA becomes integrated into the host cell genome by means of the viral integrase enzyme. The integrated provirus will irreversibly persist in the cell throughout its life and it will be transmitted to daughter cells following mitosis. After integration, the host cell will use the RNA polymerase II transcription machinery to stably express the proviral genes from the 5′-LTR and to transcribe the full-length genomic RNA. Thus, progeny virus particles can be packaged and released out of the infected cell by budding at the plasma membrane, where newly synthesized viral envelope glycoprotein units have been previously incorporated.

## 4. From Non-Primate Lentivirus to Lentiviral Vector

The main goal that concerns development of a lentiviral vector is to remove unnecessary and potentially harmful sequences from the parental lentivirus genome, while conserving a high viral titer. Substantial advances in understanding of lentivirus life cycles and their various constituent proteins allowed the bioengineering of replication-deficient lentiviral expression vectors that maintain the favourable properties of parental viruses. In particular, lentiviral vectors retain the nuclear import mechanism that permits efficient transduction of non-dividing cells and are powerful vehicles for expressing different reporter and therapeutic constructs in dividing and non-dividing cells, as well as whole organisms. In this respect, delivery of transgene constructs may be directly in vivo or in cultured cells that are then transplanted in the individual body. Moreover, lentiviral vectors can be used when a permanent modification of the target cell is required because the integration process into the host genomic DNA provides stable expression of their transgene cargo [57].

Engineering of non-primate lentiviral expression systems capable to transduce a broad range of target cells followed the general design and state-of-the-art of the HIV-1 lentiviral vector technology [58]. The rationale of these protocols is to separate the *cis*-acting sequences involved in the transfer of the viral genome to target cells, from the sequences encoding the *trans*-acting viral proteins. This “split-genome” strategy results in at least three separate expression cassettes for the so-called transfer, packaging and envelope plasmids, respectively.

The transfer plasmid carries the transgene, which is generally under the transcriptional control of an internal promoter, the viral encapsidation signal for selective packaging of the transfer plasmid RNA, and the minimal elements necessary for reverse-transcription, cDNA integration, and nuclear export of the transfer plasmid RNA (such as the rev-responsive element, RRE). Additional elements derived from other viruses may also be incorporated in the transfer vector backbone to efficiently enhance transgene expression (for example, the internal promoter from cytomegalovirus, CMV, and post-transcriptional regulatory elements from woodchuck hepatitis virus, WPRE) or to allow simultaneous expression of two transgenes from a single internally promoted transcript (for example, the internal ribosomal entry site, IRES, from picornavirus) [59,60]. 

The packaging plasmid contains the *gag* and *pol* genes encoding both the structural and enzymatic proteins of the virion particle and may include the viral gene coding for the Rev protein, which is essential for post-transcriptional transport of the viral mRNAs from nucleus to cytoplasm. Finally, the envelope plasmid provides a heterologous glycoprotein derived from other enveloped viruses, in order to re-direct or expand tropism towards a wide range of cell types, and to increase the infectivity and stability of vector particles. Although glycoproteins from rabies virus, mokola virus, and gamma-retroviruses have been used, vesicular stomatitis virus glycoprotein (VSVG) remains, so far, the most widely used glycoprotein for pseudotyping of lentiviral vectors [61,62,63].

Importantly, during the lentiviral vector production process, the replication-defective viral particles produced are capable of transferring two copies of plus-stranded RNA encoding the transgene of interest to the target cell, but are limited to this single round of the infection process without spreading. Furthermore, the current minimal lentiviral vector systems lack all viral auxiliary genes that do not play important roles in viral transduction, since many of these genes have been shown to be detrimental to cell survival [64]. Altogether, these precautions minimize the possibility of a replication-competent lentivirus (RCL) being generated via recombination in vivo [65]. Such a possibility is highly unlikely because of multiple safety measures adopted during vector production: basically, all the components are supplied in trans, self-inactivating LTRs are used, and the plasmids used show limited regions of homology each other. However, the theoretical risk exists and an RCL assay is usually required to confirm the lack of any replicating virus prior to the vector being used clinically.

To date, almost all non-primate lentivirus species have been engineered into efficient lentiviral vectors following the protocol described above [66,67,68,69,70]. Regarding the RCL assay, in principle it is not strictly required for these particular vectors, since they would neither come in to contact with the wild type virus into a human, nor they could be spread in to the environment due to rapid inactivation by the complement system.

Historically, FIV has been the first engineered non-primate lentivirus, and it is still now considered an attractive alternative to the primate lentiviral vectors for gene therapy. In this regard, it should be emphasized that the highly restricted natural FIV tropism acts as a double-edged sword. On one side, it constitutes a potentially important biosafety feature for clinical application, since FIV does not infect humans despite extensive exposure [71,72,73]. On the other hand, it represents an obstacle for adapting FIV to gene transfer in human cells. Pseudotyping by the incorporation of heterologous envelope proteins into the FIV viral particle provided the opportunity to redirect the tropism towards different target cells and tissues. For example, the pantropic VSVG envelope protein allowed the preparation in human cells of FIV-derived vectors that efficiently transduce human, feline, and murine targets, showing minimal cytotoxicity [62,66,74]. However, pseudotyped FIV particles are still subject to innate antiviral restrictions put in place by the transduced cells. In particular, the TRIM5α (tripartite motif-containing 5 α) protein and its species-specific variants were found to account for a post-entry blocking activity by binding to viral capsid protein trimers and, in turn, disable the incoming viral particles prior to reverse transcription of the viral genome [75,76]. It follows that in gene therapy applications associated to very low multiplicity of infection such a post-entry restriction may drastically reduce the efficiency of FIV-derived vectors [77]. In this regard, titration of TRIM5α proteins could be accomplished by increasing the lentiviral vector dosage or by adding genome-less pseudotyped virus-like particles. On the other hand, the existence of the mentioned TRIM5α-dependent mechanism could provide an innate defence against systemic propagation of non-primate lentiviral particles that might theoretically arise in gene therapy. Another block to the completion of the wild-type FIV life cycle consists in the lack of long terminal repeat (LTR)-driven transcription in human cells. This drawback has been in large part overcome by replacing the U3 promoter region of the FIV LTR with a stronger enhancer/promoter [66,78], thereby bypassing the hazards of vector manufacturing in feline producer cells.

Soon after the development of FIV-based vectors, recombinant EIAV lentiviral vectors were engineered following the “split-genome” strategy described above [67,79]. The biosafety of this platform has been improved by engineering self-inactivating vectors in which part of the viral promoter situated in the LTR was deleted. In the latest generation of EIAV minimal vectors, the *gag* and *pol* genes have been codon-optimized, providing an additional safety advantage that makes homologous recombination highly improbable [80].

## 5. Non-Primate Lentiviral Vectors for Ocular Gene Therapy

Studies in the mammalian eye have used mainly three distinct viral vector systems to transfer foreign genes into post-mitotic retinal cells. Among these, adenoviral (AdV) vectors probably represent the third choice in order of importance, essentially because they usually mediate transient transgene expression and induce cell-mediated immune response [81,82]. Recombinant adeno-associated viral (AAV) vectors overcome these disadvantages, achieving efficient transduction of retinal ganglion cells and, to a lesser extent, photoreceptor cells [83,84]. In the former case, the transgene product undergoes anterograde axonal transport through the optic nerve, reaching the dendritic arbors at central target sites [84,85,86]. In the standpoint of gene therapy, this is certainly an adverse effect, since it could theoretically foretell dissemination of the viral particles over a long distance from the ocular district. However, an even bigger potential flaw of AAV vectors consists in the limited cloning capacity of less than 5 kb, which precludes cloning of large genes into a single viral particle [87]. Some efforts have been made towards increasing the cargo size of AAV vectors, both by the production of high-capacity AAV/AdV hybrid vectors and the improvement of trans-splicing technologies [88,89]. Nevertheless, lentiviral vectors currently continue to hold the broadest transgene-carrying capacity (of about 10–11 kb), which allows either delivery of large transgene constructs or co-delivery of multiple genes [90,91]. Such a strength, along with other facets discussed throughout this review, clearly indicates that lentiviral vectors represent a valuable system for gene transfer purposes.

In the chronology of ocular gene therapy, the first lentiviral vector evaluated was derived from the primate HIV lentivirus [92]. Although, as described, the possibility of generating replication-competent virus particles in a clinical trial from such a lentiviral vector is highly remote, psychological barriers in patients regarding use of HIV-based vectors could significantly hinder recruitment during clinical settings. Non-primate lentiviral vectors allowed to fully overcome this concern by virtue of their undoubted incapability of replicating in human cells. In addition, their relatively simple genome organization coupled to the above-mentioned transgene-carrying capacity facilitated vector development. 

To date, the toxicity, biodistribution, and shedding characteristics of FIV-, BIV- and EIAV-derived vectors have been examined intra-ocularly by several studies (Table 3), and all of them coherently showed that these lentiviral vectors are safe, well tolerated, and localized to the site of administration in the eye. In this respect, the eye is an excellent target for gene therapy applications, essentially for two main reasons: it is easily accessed by standard injection of therapeutic lentiviral vectors, and it is isolated from the rest of the body via the blood–retina barrier. These features minimize any vector dissemination from the target ocular structures, thereby lowering the risk associated with potential side effects of treatment, such as insertional mutagenesis, in non-target cell types [93,94].

The distinct anatomically compartmentalized structures of the mammalian eye allow a multitude of vector administration routes, including intra-stromal, sub-conjunctival, peri-ocular, intra-cameral, intra-vitreal, and sub-retinal administration, altogether showing high variation in terms of lentiviral vector transduction. It has been also shown that choosing the appropriate vector administration route is highly influential in targeting specific cell types. For example, intra-vitreal delivery typically fails to achieve efficient transduction of retinal cells, possibly due to at least three known aspects. In brief, the intra-vitreally delivered lentiviral vector is diluted immediately on mixing with the vitreous humour and is situated outside the immune-privileged retinal compartment, being at increased risk for neutralization [117,118]. Moreover, the retinal internal limiting membrane and the posterior hyaloid face of the vitreous cortex act as physical barriers to vector diffusion across the eye subcompartments [119]. In contrast to intra-vitreal delivery, injection of VSVG-pseudotyped recombinant lentiviral vectors into the anterior chamber results in efficient and stable transduction of the corneal endothelium together with the trabecular meshwork. Sub-retinal injection instead appears to transduce cells of the retinal pigment epithelium along with neuronal and glial cells of the neuroretina to a variable extent. In this case, the distribution of transduced cells invariably remains focused mostly near the injection site [102,120]. Several findings obtained on different animal host species pointed out that this occurrence is not dependent on the degree of maturity of the retina, but rather relies upon direct lentiviral vector access to cells injured by the injection procedure [102,121]. In general, sub-retinal injection is the most used route of vector administration and it is currently used for human trials (see below).

Importantly, controlled comparison studies evaluating the ability of primate and non-primate-derived lentiviral vectors to transduce human and rabbit corneas, as well as human perfused eyes and subretinally-injected monkey eyes, established that FIV- and EIAV-based vectors are equivalently or even more efficacious, respectively, relative to HIV vectors [95,96,97,100]. Compelling evidence also showed that the high transduction aptitude of EIAV-based vectors extends to human corneal endothelial cells after the cryopreservation of ex vivo corneas, with no evidence of cellular dysfunction [104,122]. This finding is of prominent interest for future therapeutic application to treat corneal endothelial disorders. In fact, rather than use one cryopreserved cornea for a single recipient patient, EIAV-transduced and cultured endothelial cells could be theoretically transplanted as donors to multiple patients. In this regard, the advancements obtained during the last decade anticipate a revolutionary shift in the treatment of corneal diseases by means of the selective transplantation of components of the cornea instead of full-thickness keratoplasty, with improved recovery times and visual outcomes [123]. In light of this, transplantation of transduced endothelial cells could further implement a minimally invasive treatment with a lower risk of rejection than traditional corneal transplantation.

As evidenced throughout the specialized literature, bioengineering of non-primate lentiviral vectors involved the inclusion of distinct promoter sequences to direct appropriate transgene expression. In principle, adoption of a ubiquitous promoter, such as the cytomegalovirus immediate early gene promoter (CMV), should guarantee constitutively high and continuous transgene expression [124,125]. Contrary to this expectation, several studies highlighted that, following integration into the host cell genome, a given lentiviral transgene exhibits variegated expression and extinguishes its expression sooner or later [126,127,128]. Such a propensity for transgene silencing, termed the chromosomal position effect, reflects the not-permissive chromatin environment at the genomic insertion site, resulting in the spreading of heterochromatin into the vector sequences [129,130,131]. A growing body of evidence showed that specialized nucleoprotein complexes known as chromatin insulators can minimize chromosomal position effects when these regulatory elements are incorporated into the transferred unit [132,133,134,135,136]. Studying chromosomal position effects is extremely laborious, since it would require the establishment of a series of clonal lines each harbouring a single randomly integrated lentiviral vector, followed by the systematic mapping of each integration site, as well as the long-term vector expression in each cell clone. Although so far similar studies have not been carried out in the mammalian eye or in cells derived from ocular districts, the direct role of chromosomal position effects deserves to be better investigated in the future.

Tissue-specific promoters would be expected to be relatively weak compared to their constitutive counterparts, essentially due to the presence of *cis*-regulatory elements that preclude promoter activity in non-target cell types. Surprisingly, however, an effective increase in photoreceptor expression has been reported using HIV-based vectors in which the CMV promoter was substituted with the photoreceptor-specific *rhodopsin* promoter [92,137]. Similar results have been described following sub-retinal injection in mice of EIAV-based vectors bearing the GFP reporter under the control of photoreceptor-specific promoters in combination with an enhancer element isolated from the *interphotoreceptor retinoid-binding protein* gene [103]. A relevant clinical implication of these findings consists in maintaining high transgene expression levels, while hampering the possibility of undesirable transduction of non-target retinal cells at the site of lentiviral vector administration.

## 6. Most Recent Advancements and Future Directions

For the variety of reasons explained in the previous section, all of the lentiviral vectors standing at the forefront of gene therapy in ophthalmology are based on EIAV. Currently there are three clinical trials that use EIAV lentiviral vectors (namely Stargen, Ushstat, and Retinostat) for treatment of distinct eye diseases (Table 3). StarGen contains the wild type *ABCA4* gene, which encodes for a photoreceptor-specific member of the ABC transporter family [112,138,139]. In the worldwide population, *ABCA4* is susceptible to frequent mutations that cause the juvenile cone-rod dystrophy called Stargardt disease [140,141]. Although *ABCA4* is a quite large and complex gene, the corresponding *ABCA4* cDNA, of about 7 kb, has been comfortably cloned into the StarGen vector, which efficiently corrected the disease phenotype in *ABCA4*^−/−^ knockout mice [107]. More recently, a GLP toxicology study described subretinal injection in rabbits and macaques of a StarGen reporter vector, providing additional encouraging elements for utilization of this non-primate lentiviral vector to treat patients affected by Stargardt disease [112]. 

UshStat vector has been obtained by cloning the human *myosin VIIa* gene, encoding a non-conventional myosin motor protein, under the control of the CMV promoter in an EIAV-based vector [115]. Mutations in the mentioned gene are commonly associated with the onset of the retinal degenerative disease named Usher syndrome type IB [142]. The potential of UshStat for gene therapy purpose has been recently supported by functional restoration of phenotype in the *shaker1* mouse model of Usher syndrome, following subretinal administration of UshStat [115]. Importantly, both StarGen and UshStat vectors have been licensed to Sanofi and are SAR422459 and SAR421869, respectively. 

Last, but not least, RetinoStat has been developed to tackle retinal neovascularization, which is associated to the age-related macular degeneration. Neovascularization is quenched physiologically by the angiostatic proteins expressed during the process of normal wound healing [143]. Based on this concept, RetinoStat contains two potently angiostatic genes, *endostatin* and *angiostatin* [111]. When subretinally injected in mice, rabbits, and macaques, RetinoStat efficiently abolished the retinal blood vessel proliferation [102,108,111]. This evidence opened the way for the first phase I clinical trial testing the safety of subretinal injection of a non-primate lentiviral vector in patients affected by neovascular age-related macular degeneration [116]. This study confirmed that not only RetinoStat provides robust and sustained expression of the bicistronic transgene, but it also helps to reduce the excess fluid characteristically residing in the neural retina of sick patients [116]. 

A complementary non-primate lentiviral platform, named EncorStat, has been more recently developed to minimize corneal neovascularization, which represents the most important risk factor for rejection following corneal transplantation [144]. To this purpose, the bicistronic organization of EncorStat allows the delivery of the same two angiostatic proteins synthesized by Retinostat [113]. Worth mentioning, rabbit corneas incubated with EncorStat before surgery significantly suppressed neovascularization in a rabbit model of corneal rejection, favouring the improvement of an ex vivo therapeutic protocol [113].

RetinoStat and EncorStat are only two recent examples testifying that the carrying capacity of lentiviral vectors may permit co-delivery of multiple genes. This aspect is of relevant interest for future clinical application, since it could facilitate multifaceted targeting of eye disorders with potentially greater therapeutic effects than single gene delivery approaches. Furthermore, beneficial outcomes could be extended to cell types not directly transduced by lentiviral vectors if, for example, a paracrine effect could be attained by a sufficient amount of a therapeutic protein secreted by adjacent transduced cells.

Beyond speculative aspects about possible future application of non-primate lentiviral vectors, all the studies described in this section allow to outline two general points: (1) most ocular diseases actually lack effective gene therapies, and (2) there is a major need for animal models that closely mirror the human eye pathologies. Although relevant information on the latter issue can be obtained from human epidemiological studies, the use of suitable animal models is critical in identifying risk factors for a given ocular disease. In addition, animal models represent the most commonly used experimental tool to obtain a more comprehensive understanding of the basic molecular mechanisms that drive disease progression, also paving the way for evaluation of the biosafety and efficacy of therapies, including those based on the use of non-primate lentiviruses. In the last decades, multiple animal models mimicking eye diseases have been developed, each of them owning unique peculiar features [145]. Rodents, such as mice and rabbits, have been and probably will continue to be the most commonly used mammalian models for vision research, due to the fact that they are relatively easy to house and manipulate. However, the visual systems of rodents lack some anatomical and functional features found in humans: for example, they do not have a macula [146], the region of fine visual discrimination affected by human retinal neovascular diseases [147,148].

Using of non-human primates for screening to detect potential therapeutics allows overcoming this problem, with more direct relevance to human conditions. On the other hand, the value of employing primates in scientific research must be carefully evaluated due to challenging expense and the necessity for highly specialized personnel and equipment. In fact, it is often preferable to perform pilot experiments in evolutionary simpler organisms before making the pivotal studies in non-human primates. In this respect, zebrafish (*Danio rerio*) recently emerged as a powerful model system for studying ophthalmological disorders and for developing potential therapies to combat them. The strength of zebrafish lies primarily in the fact that the fish’s visual system mirrors the human anatomical and functional situation better compared with other animal models. Indeed, contrary to mice that have rod-dominated vision, both zebrafish and humans have cone-dominant vision and comparable cone density [149,150,151]. Several human ocular diseases affecting, among others, the corneal endothelium, lens, photoreceptors, and retinal vasculature, have already been modelled in zebrafish [152,153,154,155,156]. Compared to other animal models, zebrafish can be maintained relatively cheaply in laboratory, and have high fecundity as well as a short life cycle, allowing rapidity and high statistical power for downstream experimental procedures [157]. Furthermore, intra-vitreal and/or retro-orbital injection could be performed easily in the zebrafish eye to deliver therapeutic vectors or heterologous cells transduced ex vivo. By virtue of all these strengths, it could be reasonably anticipated that the zebrafish model will play a central role in future research on different aspects of ophthalmology and vision science, including the use of non-primate lentiviral vectors for gene therapy.

## Figures and Tables

**Table 1:** Non-primate lentiviruses and their host tropism.

Non-Primate Lentivirus	Natural Host	Historical References
visna-maedi virus (VMV)	Sheep	[13,14]
caprine arthritis encephalitis virus (CAEV)	Goat	[15,16]
equine infectious anaemia virus (EIAV)	Horse	[17,18]
feline immunodeficiency virus (FIV)	Cat	[19,20]
bovine immunodeficiency virus (BIV)	Cattle	[21,22]
Jembrana disease virus (JDV)	Bali cattle	[23,24]

**Table 2:** Differential distribution of accessory genes among non-primate lentivirus genomes.

Accessory Gene	Non-Primate Lentivirus					
	VMV	CAEV	EIAV	FIV	BIV	JDV
*rev*	+	+	+	+ ^a^	+	+
*vif*	+	+	-	+	+	+
*tat*	+ ^b^	+ ^b^	+	-	+	+
*orfS*	+	+	+	+ ^c^	-	-
*vpy/vpw*	-	-	-	-	+	-
*tmx*	-	-	-	-	+	+
*s2*	-	-	+	-	-	-

^a^ The Rev protein of FIV bears a divergent non-consensus nuclear export signal; ^b^ Tat proteins from VMV and CAEV lack the transactivation function; ^c^ *orfS* gene of FIV is called *orf2.*

**Table 3:** Overview of studies using non-primate lentiviral vectors for ocular gene delivery.

Non-Primate Lentiviral Vector	Target Host Cell/Tissue/Organism	Delivered Genes	References
FIV	Perfused human eyes	*lacZ, eGFP*	[95,96]
FIV	Macaque	*lacZ*	[97]
FIV	Mouse	*lacZ, β-glucuronidase*	[98]
BIV	Mouse	*eGFP*	[99]
EIAV	Rabbit and human corneas, murine corneal endothelial cells	*lacZ, eGFP*	[100]
FIV	Rabbit, rat	*lacZ*	[101]
EIAV	Mouse	*eGFP*	[102]
EIAV	Mouse	*lacZ*	[103]
EIAV	Cryopreserved primary cultured human corneal endothelial cells	*eGFP*	[104]
FIV	Mouse retinal progenitor cells	*YFP*	[105]
FIV	Cat	*eGFP/myocilin ^a^, lacZ*	[106]
EIAV	Mouse	*lacZ, human ABCA4*	[107]
EIAV	Mouse	*endostatin/angiostatin ^a^*	[108]
FIV	Macaque	*eGFP*	[109]
FIV	Rabbit	*NBCe1-shRNA/copepod-GFP ^a^*	[110]
EIAV (RetinoStat)	Rabbit, macaque	*endostatin/angiostatin ^a^*	[111]
EIAV (StarGen)	Rabbit, macaque	*ABCA4*	[112]
EIAV (EncorStat)	Rabbit, primate and human corneal tissue	*endostatin/angiostatin ^a^*	[113,114]
EIAV (UshStat)	Mouse, macaque	*myo7A*	[115]
EIAV (RetinoStat)	Human patients	*endostatin/angiostatin ^a^*	[116]

^a^ the indicated transcription units were co-delivered from lentiviral vectors bearing bicistronic transgenes.
